# Draft Genome Sequences of Klebsiella pneumoniae Strains Isolated from Immunocompromised NOD-scid Gamma Research Mice

**DOI:** 10.1128/MRA.00869-19

**Published:** 2019-10-17

**Authors:** Anthony Mannion, Niora Fabian, Melissa Stair, Jody Dzink-Fox, Sebastian E. Carrasco, Ellen Buckley-Jordan, Damodaran Annamalai, James G. Fox

**Affiliations:** a Division of Comparative Medicine, Massachusetts Institute of Technology, Cambridge, Massachusetts, USA; University of Maryland School of Medicine

## Abstract

Thirteen Klebsiella pneumoniae isolates cultured from feces, intestines, liver, lungs, and blood from immunocompromised NOD-scid gamma (NSG) mice with clinical illness, housed at a biomedical research institute, were sequenced using Illumina MiSeq technology for elucidation of pathogenic potential and genes encoding antibiotic resistance.

## ANNOUNCEMENT

Klebsiella pneumoniae is a gastrointestinal opportunistic pathogen that is responsible for septicemia, urinary tract infections, and pneumonia in immunocompromised hosts (1–3). NOD-scid gamma (NSG; NOD.Cg-*Prkdc^scid^ Il2rg^tm1Wjl^*/SzJ) mice are immunocompromised due to defective immune cell development/function and are used as cancer xenograft, humanized, and infectious disease models (4). NSG mice from a closed breeding colony housed at an Association for Assessment and Accreditation of Laboratory Animal Care (AAALAC)-accredited biomedical research institute experienced unexpected diarrhea, morbidity, and mortality. K. pneumoniae was isolated from feces, intestines, liver, lungs, and blood via aerobic and anaerobic cultures from male and female mice (juvenile to 6 months) with bronchopneumonia, bacteremia, and/or normal colonic content that were incubated overnight at 37°C. While K. pneumoniae can cause illness in laboratory mice (5), the literature lacks genomic characterization of mouse isolates. Therefore, genomes from 13 representative isolates cultured from NSG mice, described above, were sequenced for characterization of pathogenic potential and mechanisms of antibiotic resistance.

Aerobic cultures grown overnight in LB medium at 37°C were pelleted for genomic DNA purification using the Roche High Pure PCR product purification kit. Barcoded libraries were constructed using the QIAseq FX DNA library kit and sequenced with an Illumina MiSeq instrument (2 × 300-bp reads). Raw sequence reads were decontaminated of adapters and quality trimmed using BBDuk (v38.34; parameters were ktrim=r, k=23, mink=11, hdist=1, tpe, tbo, qtrim=rl, trimq=10, qin=33) for *de novo* contig assembly with SPAdes (v3.10.0) and genome annotation by Rapid Annotations using Subsystems Technology (RAST), both hosted by PATRIC (6). Draft genomes ranged from 5,392,816 to 5,416,362 bp in 113 to 152 contigs with a GC content of 57.4% and contained 5,316 to 5,358 protein coding genes, 81 to 83 tRNA genes, and 7 to 9 rRNA genes (Table 1).

**TABLE 1:** Summary genome statistics^*a*^

Isolate accession no.^*b*^	Isolation tissue	No. of contigs	*N*_50_ (bp)	Coverage (×)	Genome size (bp)	GC content (%)	Predicted no. of:			Total no. of reads		GenBank accession no.	SRA accession no.
							Proteins	tRNAs	rRNAs	Before quality control with BBDuk	After quality control with BBDuk		
1812100012 (API 5205773)	Lung abscess	121	197,616	64.5	5,413,009	57.4	5,345	84	8	1,111,942	1,105,542	SULO00000000	SRR9209163
1812040001 (API 5215773)	Lung abscess	120	197,616	98.4	5,413,618	57.4	5,329	84	9	3,430,076	3,412,908	SULP00000000	SRR9209165
1812040001 (API 5205773)	Lung abscess	129	175,815	61.5	5,410,114	57.4	5,342	83	8	1,423,232	1,406,434	SULQ00000000	SRR9209164
1811300002 (API 5215773)	Liver	113	178,561	26.0	5,393,826	57.4	5,316	81	8	766,968	763,734	SULR00000000	SRR9209167
1811260006 (API 7215773)	Lung abscess	134	168,805	49.3	5,416,362	57.4	5,358	83	7	1,136,710	1,131,714	SULS00000000	SRR9209166
1811130035 (API 5215773)	Blood	123	178,561	60.0	5,413,798	57.4	5,341	83	7	2,582,096	2,576,930	SULT00000000	SRR9209169
1811130035 (API 5205773)	Blood	124	178,561	88.4	5,415,341	57.4	5,351	82	7	3,019,934	3,005,542	SULU00000000	SRR9209168
1811130034 (API 5215773)	Cecum	120	183,665	107.9	5,413,047	57.4	5,337	83	8	1,673,918	1,664,168	SULV00000000	SRR9209171
1811130034 (API 5205773)	Blood	119	181,707	36.2	5,403,097	57.4	5,325	81	7	1,601,638	1,592,150	SULW00000000	SRR9209170
1811130032 (API 7205773)	Blood	124	195,879	112.8	5,415,341	57.4	5,334	82	9	725,376	722,526	SULX00000000	SRR9209171
1811130032 (API 5215773)	Blood	113	197,616	25.0	5,401,953	57.4	5,320	82	8	1,732,958	1,726,996	SULY00000000	SRR9209172
1808200021	Blood	122	203,835	45.2	5,392,816	57.4	5,319	83	8	3,332,466	3,312,784	SULZ00000000	SRR9209175
1808200001	Feces	116	198,495	41.1	5,409,006	57.4	5,333	83	8	1,938,490	1,933,098	SUMA00000000	SRR9209174

a For all isolates, 2 × 300-bp sequencing was used.

b API, analytical profile index, a panel of biochemical tests used for the identification and differentiation of Gram-negative bacteria.

**FIG 1:**
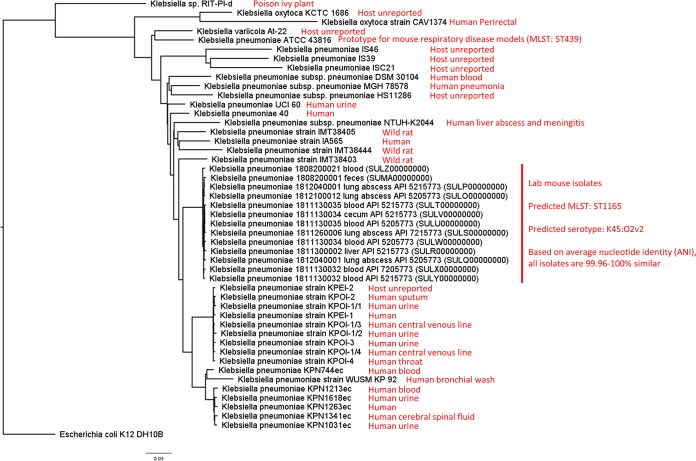
Pan-genome phylogenetic analysis of K. pneumoniae laboratory mouse isolates versus Klebsiella spp.

In phenotypic and bioinformatic analyses (with default parameters unless otherwise stated), K. pneumoniae isolates were determined to be “classical” (opportunistic pathogens that typically encode antibiotic resistance) and not “hypermucoviscous/hypervirulent” (expressing a mucoid phenotype, K1/K2 hypercapsule antigens, and virulence factors genes like colibactin) (1). Pangenome phylogenetic analysis using the Bacterial Pan Genome Analysis (BPGA) tool (v1.3.0) (7) placed all isolates in a separate clade, with genome isolates from human urine, blood, throat, and sputum as neighbors (Fig. 1). Average nucleotide identities calculated with JSpeciesWS (8) were 99.96 to 100% similar among all genomes. All genomes had a multilocus sequence type (MLST) of 1165 (ST1165), predicted using MLST 2.0 (9), and a capsule K and lipopolysaccharide (LPS) O antigens of K45:O2v2, predicted using Kaptive (v0.6.0) (10). BLASTP analysis against the Virulence Factors Database (VFDB) (11) was performed to identify virulence factors (identity, ≥90%; coverage, ≥60%). No genomes encoded *rmpA* or *magA*, either of which is required for the hypermucoviscosity phenotype. Likewise, no strains exhibited mucoid phenotypes according to negative string test results. Colibactin genes were not present in any genome. All genomes encoded the siderophore enterobactin for iron acquisition and type 1 and 3 fimbriae for adhesion and biofilm formation. Using ResFinder v3.1 (12), antibiotic resistance genes for beta-lactams and fosfomycin were predicted in all genomes. Interestingly, plasmid-encoded class 1 integrons encoding resistance against aminoglycosides, chloramphenicol, and trimethoprim-sulfonamide were identified in 12/13 genomes. Resistance to beta-lactams and trimethoprim-sulfonamide was confirmed by MIC broth assay or Etest strips.

In conclusion, K. pneumoniae isolates from NGS mice represent classical strains with pathogenic potential. The expression of plasmid-encoded multidrug resistance raises the possibility of spreading antibiotic resistance within animal research facilities and to personnel working with infected mice.

### Data availability.

Genomes have been deposited in GenBank under the following accession numbers: SULO00000000, SULP00000000, SULQ00000000, SULR00000000, SULS00000000, SULT00000000, SULU00000000, SULV00000000, SULW00000000, SULX00000000, SULY00000000, SULZ00000000, and SUMA00000000. Sequencing reads have been deposited in SRA under the following accession numbers: SRR9209174, SRR9209168, SRR9209169, SRR9209167, SRR9209163, SRR9209175, SRR9209170, SRR9209166, SRR9209171, SRR9209172, SRR9209165, SRR9209164, and SRR9209173.
